# Editorial: Optimizing sport and event tourism: enhancing destination value and addressing social issues

**DOI:** 10.3389/fspor.2026.2001351

**Published:** 2026-10-29

**Authors:** Wadih Ishac, Jae Wong Kang

**Affiliations:** 1 Department of Sport Management, College of Sport Sciences, Qatar University, Doha, Qatar; 2 Department of Business & Management (Sports Management), International College of Management, Sydney (ICMS), Manly, NSW, Australia

**Keywords:** destination value, event legacy, event tourism, resident perceptions, social sustainability, sport tourism

Sport and event tourism are increasingly assessed against expectations that extend beyond visitor arrivals and short-term expenditure. Destinations try to achieve memorable experiences, boost visitor experience to revisit, practical tourism businesses, animated public spaces, and enduring cultural and economic value. At the same time, sport events and tourism development may result with an increase of pressures relating to crowding, visitor conduct, uneven distribution of benefits, environmental management, housing affordability, and community consent. For instance, the first group from our empirical studies clarifies how value is formed within the visitor experience. Wang and Cao examine the way tourist perceived immersion is associated with tourist participation and citizenship behavior through emotional arousal. Their findings suggest that visitors can become active contributors to an experience. In sport-event settings, Ma and Han found, among participants in the 2024 Beijing Marathon, that experiential satisfaction fully mediates the association between event brand perception and revisit intention, in contrast the parallel pathway through emotional connection was not statistically significant. Similarly, Omonova et al., in a Brief Research Report that focuses on assessing the relationship between event quality and tourist satisfaction (SAT), economic outcomes, and behavioral intentions among visitors at a Silk Road heritage, shows that SAT mediates the relationships between event quality, behavioral intentions, and economic outcomes. Taken together, these studies indicate that destination value is not produced by branding, event delivery, or immersion alone; it is shaped by how visitors evaluate and participate in the experience. Furthermore, Ma et al. examine risk perception both as a threat and attraction among mountain outdoor sport tourists and report that risk perception can contribute positively to behavioral intentions directly and through experience quality, although this pattern differs by gender in their sample.

The second group of studies shifts the focus from individual experiences to the social factors that can enhance or limit a destination's value. For instance, Zhang et al. assess why tourists misbehave when traveling away from home using behavioral experiments and EEG evidence, their results shows that non-local contexts increase misbehavior intention by weakening informal social control through an abstract mindset, whereas local contexts strengthen cognitive control and normative sensitivity, thereby reducing misbehavior. In a different setting, Zhang et al. evaluate how local residents' perception of economic, image, and spatial impacts shape their support for urban marathons. There findings show that perceived economic and urban image impacts influence residents' support intentions indirectly through their attitudes. While perceived spatial impacts show both direct and indirect associations. These findings reinforce a basic point for destination planning: resident support is neither a public-relations outcome nor a permanent resource. It depends on how people experience the economic, symbolic, and spatial consequences of events in their own communities.

Heritage and spatial development further extend this argument. The systematic review by Rangkuti et al. exploring the intersection of cultural and heritage and sport tourism found, that While sports heritage integration boosts regional economies and cultural identity, sustainable management requires explicit safeguards to prevent over-commercialization, spatial crowding, and local housing/affordability pressures. At a broader geographical scale, Zhao and Han examined the spatial distribution and aggregation characteristics of STPs across china and report marked regional imbalances, where financial institutions, general public budget expenditures, and tertiary industry operating income exert the greatest explanatory power, whereas transportation and environmental indicators are less influential.

Complementing these structural perspectives, Khalimova and Ibragimov propose the Creative Sport–Event Tourism Nexus, arguing that creative placemaking can connect sport events with local cultural production, community participation, and destination differentiation. Qodirov and Urakova advocate that sustainability planning must be incorporated from the initial phases of event bidding rather than treated as a *post hoc* consideration. Qodirov et al. propose a community-focused framework for emerging destinations that brings together community-based tourism, legacy planning, and social license to operate. Evidence from emerging destinations indicates that community benefit programs, participatory governance, and heritage integration produce greater stakeholder acceptance than infrastructure investments alone.

Two further Opinion articles focus on accommodation, an often overlooked but consequential part of the sport-tourism system. Toyirova and Juraev argue that hotels can pursue economic performance and social responsibility together through pricing, event partnerships, workforce development, community engagement, and environmental practice. Toyirova et al. similarly position sustainability-led accommodation management as a dynamic accelerator to destination competitiveness. These arguments draw attention to the fact that visitor capacity and value are partly produced through accommodation systems. They also raise questions that require stronger empirical examination: who gains from event-time revenue, who bears the costs of price increases and labor demand, and what mechanisms ensure that sustainability commitments extend beyond promotional statements?

In conclusion, the articles in this Research Topic provide a broad understanding of how sport and event tourism can strengthen destinations while also creating social challenges that need careful attention. Together, they show that destination value is shaped not only by visitor satisfaction and economic activity, but also by safety, resident attitudes, visitor behavior, cultural heritage, community participation, spatial development, and the capacity of local institutions. The studies offer useful directions for destination managers and policymakers: improve event and service quality, involve local communities, protect heritage, manage accommodation and visitor pressures, and consider sustainability from the early stages of planning. At the same time, the collection highlights the need to look beyond short-term economic results and pay greater attention to inclusion, affordability, environmental impacts, and the fair distribution of benefits. Overall, the Research Topic shows that successful sport and event tourism depends on creating experiences and destinations that are valuable not only for visitors, but also for the people and communities who live there.

